# Metabolic syndrome and carotid plaque in Chinese taxi drivers: a cross-sectional study of 5,999 participants

**DOI:** 10.3389/fpubh.2026.1812128

**Published:** 2026-06-08

**Authors:** Xinxiang Guo, Wenqi Wu, Tingting Cui, Feng Zhu, Hong Yu, Haifeng Shan

**Affiliations:** 1 Labor Model Health Management Center, Beijing Rehabilitation Hospital, Capital Medical University, Beijing, China; 2 Department of Rheumatism and Immunology, Peking University Shenzhen Hospital, Shenzhen, Guangdong, China; 3 Senior Department of Hepatology, The Fifth Medical Center of Chinese PLA General Hospital, Beijing, China; 4 Gastrointestinal Rehabilitation Center, Beijing Rehabilitation Hospital, Capital Medical University, Beijing, China

**Keywords:** cardiovascular disease, carotid plaque, metabolic syndrome, occupational health, taxi drivers

## Abstract

**Background:**

Taxi drivers face occupational exposures that are associated with cardiovascular disease (CVD) risk, including prolonged sedentary behavior, irregular meal patterns, and psychosocial stress. However, comprehensive assessments of metabolic health in this population, particularly with standardized comparisons to the general population, are limited.

**Methods:**

This cross-sectional study analyzed data from 5,999 Beijing taxi drivers undergoing annual health examinations (2022–2023). Metabolic risk factors were assessed and metabolic syndrome (MetS) was defined using the 2009 harmonized criteria. Carotid ultrasound was performed in 4,582 participants (76.37%) to detect subclinical atherosclerosis. Age-standardized prevalence ratios (SPRs) were calculated by comparing taxi drivers with the Beijing general population. A parallel occupational health survey (*n* = 5,922) from the same population characterized work conditions and lifestyle factors.

**Results:**

Compared with the Beijing general population, male taxi drivers exhibited significantly elevated metabolic risk: hypertension (SPR = 2.25, 95% CI: 2.16–2.33), obesity (SPR = 2.19, 95% CI: 2.10–2.28), carotid plaque (SPR = 2.00, 95% CI: 1.91–2.10), and fatty liver disease (SPR = 1.76, 95% CI: 1.71–1.82; all *p* < 0.001). Occupational survey data showed substantial adverse work exposures: 62.25% had worked ≥20 years as taxi drivers, 36.31% reported insufficient rest (<4 days/month), 58.17% reported difficulty accessing restrooms, and 53.31% reported meal-related difficulties during work. Among those with carotid ultrasound data, 40.77% had carotid plaque. MetS (≥3 of 5 components) was independently associated with carotid plaque (adjusted OR = 1.28, 95% CI: 1.11–1.48, *p* < 0.001). The association remained robust in inverse probability weighting (OR = 1.27, 95% CI: 1.10–1.47), modified Poisson regression (PR = 1.12, 95% CI: 1.05–1.19), and analyses using alternative central obesity definitions.

**Conclusion:**

Chinese taxi drivers bear a substantially elevated burden of metabolic disorders compared with the general population. The high prevalence of adverse occupational exposures provides important context for understanding these findings. Targeted occupational health interventions addressing lifestyle and work conditions in this population may be warranted.

## Introduction

Cardiovascular disease remains the leading cause of mortality globally, with approximately 20 million deaths per year in recent estimates (1). In addition to traditional risk factors such as hypertension, diabetes, dyslipidemia, and obesity, occupational and psychosocial exposures are increasingly recognized as important contributors to cardiovascular risk (2).

Professional drivers, including taxi and bus drivers, represent an occupational group with high cardiometabolic burden (3–5). The work environment of taxi driving typically includes prolonged sedentary posture, irregular schedules, barriers to healthy eating, psychosocial strain from traffic and passenger interactions, and chronic exposure to traffic-related air pollution (6). These coexisting occupational exposures may be associated with metabolic dysfunction and subclinical vascular injury. Several studies conducted among taxi drivers in the Middle East have provided further evidence of this elevated risk. In Yazd, Iran, Rezaei Hachesu et al. (7) reported that 89.1% of taxi drivers had at least one cardiovascular risk factor, with nearly a quarter having three or more concurrent risk factors. The same research group subsequently found a metabolic syndrome prevalence of 37.5% among male taxi drivers using ATP III criteria, with hypertriglyceridemia being the most prevalent component; notably, 40% of drivers with MetS had an intermediate 10-year cardiovascular event risk (8). In Tabriz, a cross-sectional study of 402 taxi drivers revealed that 12.4% had cardiovascular disease and 10.2% had a history of myocardial infarction, with sleep disorders and depression compounding cardiovascular risk in this population (9). Beyond metabolic outcomes, occupational pressures are also associated with risky health-related behaviors among taxi drivers, as demonstrated in a study from Bandar Abbas showing high rates of unsafe behaviors driven by income-related time pressures (10). These findings from diverse settings collectively highlight the global vulnerability of taxi drivers to cardiometabolic disorders.

**Figure 1:**
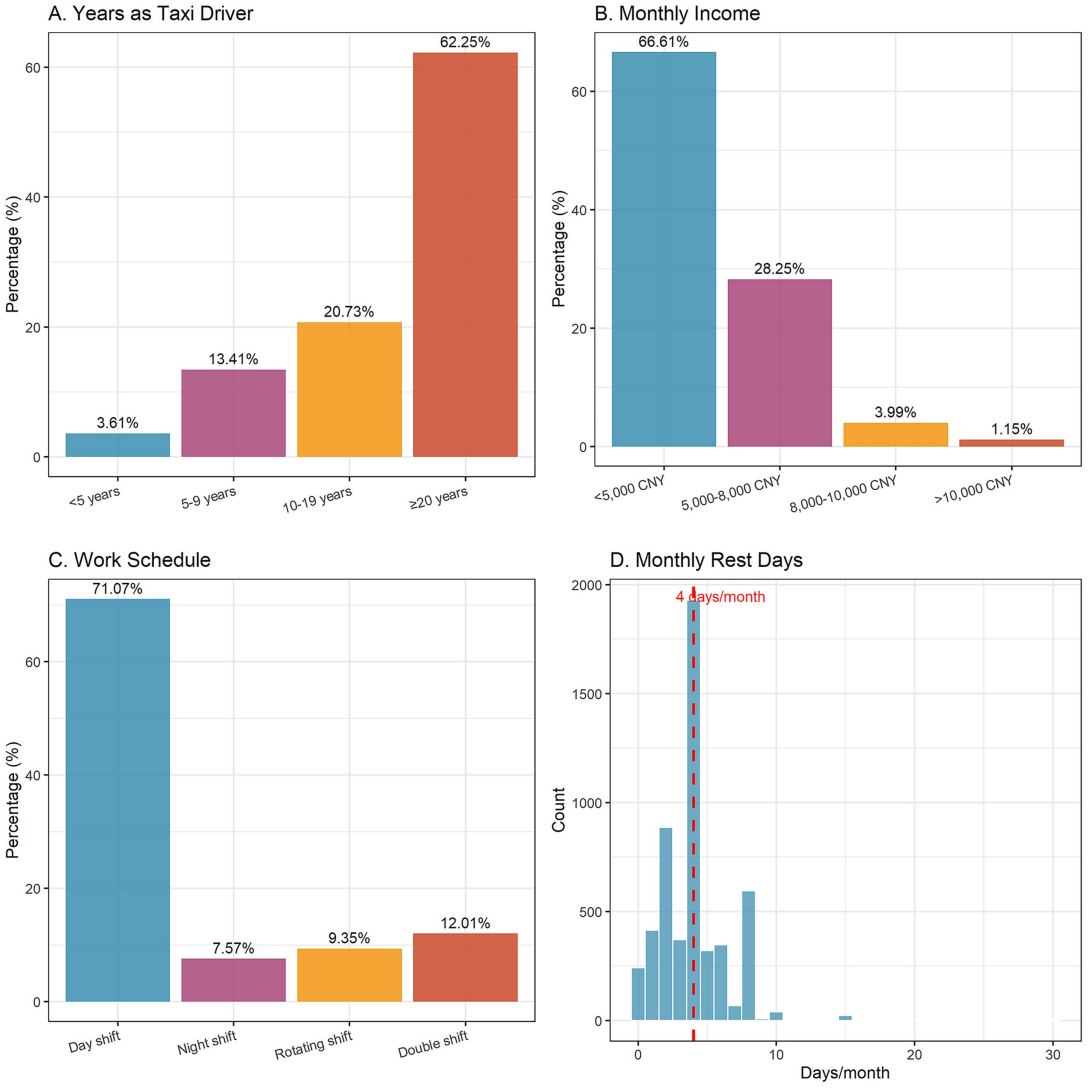
Occupational characteristics of Beijing taxi drivers (*N* = 5,922). Four-panel diagram showing: **(A)** Distribution of years working as taxi driver; **(B)** Monthly income distribution; **(C)** Work schedule patterns; **(D)** Monthly rest days distribution.

Subclinical atherosclerosis, as assessed by carotid ultrasound, provides a valuable marker of cardiovascular risk that precedes clinical events (11). Carotid plaque presence has been shown to predict future cardiovascular events more accurately than carotid intima-media thickness alone (12). While previous studies have documented elevated cardiovascular risk in professional drivers (13–15), including the studies described above, several important gaps remain in the current literature. First, most existing studies of taxi drivers have been limited by relatively small sample sizes (typically 100–400 participants), precluding robust multivariable analyses and detailed subgroup assessments. Second, few studies have employed objective measures of subclinical atherosclerosis, such as carotid ultrasound, to evaluate whether metabolic risk clustering in taxi drivers translates into measurable vascular injury. Third, age-standardized comparisons with the general population, which are essential for quantifying occupation-attributable excess risk, have rarely been performed. Finally, comprehensive assessments integrating both clinical examination data and detailed occupational exposure characterization within the same population are lacking (see Figure 1).

The present study addresses these gaps by leveraging two complementary data sources collected from the same occupational population of Beijing taxi drivers during an annual union-sponsored health program. Clinical examination data, including anthropometric measurements, laboratory tests, and carotid ultrasound, were obtained from 5,999 participants, while a parallel occupational health survey capturing work conditions, lifestyle factors, and psychosocial measures was administered to 5,922 drivers from the same population during the overlapping time period. Although individual-level linkage between datasets was precluded by the anonymized study design, both data sources reflect the health status and occupational exposures of a single, well-defined occupational cohort, enabling comprehensive population-level characterization.

This study aimed to characterize the prevalence of metabolic risk factors and subclinical atherosclerosis in taxi drivers, compare these prevalences with age-standardized estimates from the Beijing general population, evaluate associations between MetS and carotid plaque, and describe the occupational context underlying these findings.

## Materials and methods

### Study design and population

This cross-sectional study analyzed data from two parallel sources collected from Beijing taxi drivers participating in a labor union-sponsored annual health program (2022–2023). The clinical examination data cohort (*n* = 5,999) comprising anthropometric measurements, laboratory tests, abdominal ultrasound, and carotid ultrasound.

The health examination was offered free of charge to all union members; participation was voluntary. Carotid ultrasound was included as an optional screening component; 1,417 participants (23.6%) declined this voluntary examination, resulting in carotid data for 4,582 participants.

The occupational health survey data cohort (*n* = 5,922) capturing detailed information on work conditions (tenure, income, work schedule, rest patterns), lifestyle factors (smoking, alcohol, exercise, diet), sleep status, and psychosocial measures including job burnout, work stress, and job satisfaction. Due to the anonymized design required by the labor union for participant privacy protection, individual-level linkage between the clinical and survey datasets was not feasible. However, both datasets were collected from the same occupational population during overlapping time periods. The occupational survey data are therefore treated as ecological (population-level) exposure characterization rather than as individual-level predictors, and all occupational-clinical comparisons in this study should be interpreted accordingly.

Inclusion criteria for the clinical cohort were: (1) current employment as a taxi driver and (2) complete data for anthropometric measurements and at least one laboratory test. Participants with incomplete demographic data were excluded. The study was approved by the Institutional Ethics Committee (approver number: 2025bkky-040), and the requirement for informed consent was waived due to the retrospective nature of the data analysis.

### Clinical measurements

All examinations were performed by trained medical professionals following standardized protocols. Body weight and height were measured with participants wearing light clothing and no shoes, and body mass index (BMI) was calculated as weight (kg) divided by height squared (m^2^). Waist circumference was measured at the midpoint between the lowest rib and the iliac crest. Blood pressure was measured using an automated sphygmomanometer (OMRON HBP-9030) after participants had rested in a seated position for at least 5 min; two measurements were taken at 1-min intervals, and the average was recorded.

Venous blood samples were collected after an overnight fast of at least 8 h. Fasting plasma glucose (FPG), total cholesterol (TC), triglycerides (TG), low-density lipoprotein cholesterol (LDL-C), and high-density lipoprotein cholesterol (HDL-C) were measured using an automated biochemical analyzer (Canon TBA-FX8) with standardized reagents.

Abdominal ultrasonography was performed by experienced sonographers using high-resolution ultrasound systems (Mindray Resona R9T). Fatty liver disease was diagnosed based on standard ultrasonographic criteria, including increased liver echogenicity compared to the renal cortex, vascular blurring, and deep attenuation of the ultrasound signal (16). Carotid ultrasonography was performed as part of the routine annual health examination at the examination center of Beijing Rehabilitation Hospital, using linear array transducers (frequency: L9-3U). Examinations were conducted by the center’s pool of certified vascular sonographers on rotation. Carotid plaque was defined according to the Mannheim consensus criteria (17) as a focal structure encroaching into the arterial lumen by at least 0.5 mm or 50% of the surrounding intima-media thickness (IMT), or demonstrating a thickness >1.5 mm.

### Definitions of metabolic risk factors

Five metabolic risk factors were assessed. Hypertension was defined as systolic blood pressure ≥140 mmHg and/or diastolic blood pressure ≥90 mmHg, or self-reported physician diagnosis with current antihypertensive medication use (18). Elevated fasting glucose was defined as FPG ≥ 6.1 mmol/L (110 mg/dL), or self-reported physician diagnosis of diabetes with current glucose-lowering medication use (19). Dyslipidemia was defined as the presence of any of the following: LDL-C ≥ 3.40 mmol/L, TG ≥ 1.70 mmol/L, HDL-C < 1.0 mmol/L, TC ≥ 5.7 mmol/L, or current use of lipid-lowering medication (20). Overweight/obesity was defined as BMI ≥ 24.0 kg/m^2^ based on Chinese population-specific criteria (21). Fatty liver disease was identified by ultrasonographic evidence of hepatic steatosis as described above (16).

Metabolic syndrome (MetS) was defined according to the 2009 Joint Interim Statement harmonized criteria (22), requiring the presence of at least three of the following five components: (1) elevated waist circumference (≥90 cm for men, ≥80 cm for women, using Asian-specific cutpoints); (2) elevated triglycerides (≥1.70 mmol/L or drug treatment); (3) reduced HDL-C (<1.03 mmol/L for men, <1.29 mmol/L for women, or drug treatment); (4) elevated blood pressure (systolic ≥130 mmHg and/or diastolic ≥85 mmHg, or antihypertensive treatment); and (5) elevated fasting glucose (≥5.6 mmol/L or drug treatment for diabetes).

### Occupational health survey measures

The occupational health survey assessed multiple domains of work conditions and health behaviors. Work characteristics included years of experience as a taxi driver, monthly income level, work schedule type (day shift, night shift, rotating shift, or double shift), and number of monthly rest days. Work-related difficulties encompassed difficulty accessing meals during shifts, toilet access difficulties, and habitual breakfast skipping. Sleep status was evaluated through self-rated sleep quality on a four-point scale (very good, good, poor, very poor) and self-reported daily sleep duration in hours. Lifestyle factors included smoking status (never, former, or current smoker), alcohol consumption frequency, and physical exercise frequency. Psychosocial measures comprised a job burnout scale consisting of three subscales scored on a 1–7 point scale, a work stress scale with six dimensions on a 1–5 point scale, job identity measured on a 1–5 point scale, and overall job satisfaction rated on a five-point Likert scale.

### Age-standardized comparison with general population

To compare metabolic risk factor prevalences between taxi drivers and the general population, we calculated standardized prevalence ratios (SPR) using age-stratified detection rates from the Beijing Health Examination Annual Report (2022–2023) as the reference (23). Expected cases were calculated by applying age-specific reference rates (18–29, 30–39, 40–49, 50–59, ≥60 years) to the taxi driver population age structure. Ninety-five percent confidence intervals were estimated using Byar’s approximation, and statistical significance was assessed using z-tests (24).

### Statistical analysis

Statistical analyses were performed using R software version 4.5.2 (R Foundation for Statistical Computing, Vienna, Austria), with the significance level set at two-tailed *p* < 0.05. Continuous variables were assessed for normality using skewness statistics and visual inspection; normally distributed variables were expressed as mean ± standard deviation (SD) and compared using Student’s *t*-test, while skewed variables were presented as median (interquartile range [IQR]) and compared using the Mann–Whitney U test. Categorical variables were expressed as frequencies (percentages) and compared using the Chi-square test.

For independent risk factor analysis, multivariable logistic regression was performed for each metabolic condition separately, adjusting for age, sex, BMI, smoking status, and alcohol consumption, with results presented as adjusted odds ratios (ORs) with 95% confidence intervals (CIs). For the primary association between MetS and carotid plaque, MetS was defined using the 2009 Joint Interim Statement harmonized criteria (≥3 of 5 components), and logistic regression models were adjusted for age, sex, smoking status, and alcohol consumption. The number of MetS components was categorized as 0, 1, 2, 3, 4, and 5, using 0 components as the reference group. To assess the dose–response relationship, the number of MetS components was modeled as a continuous variable to test for linear trend (P for trend). To evaluate potential effect modification by smoking status, we included a multiplicative interaction term (MetS × smoking) in the model and reported the P for interaction. Stratified analyses were performed by age (<50 vs. ≥50 years), sex, and smoking status to examine the consistency of the primary findings; within each stratum, covariates constant across participants (e.g., sex in sex-stratified models) were excluded to avoid collinearity, and results were visualized using forest plots.

Several sensitivity analyses were performed to assess the robustness of the primary findings. Given the high prevalence of carotid plaque, we performed modified Poisson regression with robust standard errors to estimate prevalence ratios (PRs), which provide a less biased measure of relative risk when outcome prevalence exceeds 10% (25). To address potential selection bias due to missing outcome data (23.6%), we conducted inverse probability weighting (IPW) analysis; weights were derived from a logistic regression model predicting complete outcome data based on baseline characteristics, and the stability of weights was verified (Supplementary materials). We also assessed potential non-linear associations using restricted cubic splines with 3 knots. E-values were calculated to quantify the minimum strength of association that an unmeasured confounder would need to have with both the exposure and outcome to fully explain the observed association (26). Additionally, we repeated the primary analyses using alternative exposure definitions, including waist circumference-based and BMI-based obesity criteria within the MetS definition, to evaluate the sensitivity of results to definitional choices. Multicollinearity was evaluated using variance inflation factors (VIF). Additional methodological details are provided in the Supplementary materials.

Carotid ultrasonography was offered as a voluntary screening item during the health examination. Participants who did not undergo carotid ultrasound (*n* = 1,417; 23.6%) voluntarily declined this examination; their characteristics were compared with included participants. For covariates, missing rates were minimal (<2%) and were handled using available-case analysis.

## Results

### Baseline characteristics

A total of 5,999 taxi drivers were included in the final analysis, comprising 5,195 males (86.60%) and 804 females (13.40%). The mean age of the cohort was 48.8 ± 7.5 years. Baseline characteristics stratified by sex are presented in Table 1. Compared to female drivers, males exhibited significantly higher BMI (27.7 vs. 26.2 kg/m^2^), waist circumference (94.7 vs. 82.0 cm), and blood pressure levels (all *p* < 0.001).

**Table 1:** Baseline characteristics stratified by sex.

Characteristic	Overall *N* = 5,999^1^	Male *N* = 5,195^1^	Female *N* = 804^1^	*p*-value^2^
Age (years)	48.8 (7.5)	49.3 (7.4)	45.6 (7.8)	<0.001
BMI (kg/m^2^)	27.5 (4.2)	27.7 (4.1)	26.2 (4.5)	<0.001
Waist circumference (cm)	93.0 (10.7)	94.7 (9.8)	82.0 (10.3)	<0.001
Systolic BP (mmHg)	131.9 (18.1)	132.8 (17.8)	125.8 (19.1)	<0.001
Diastolic BP (mmHg)	82.5 (12.4)	83.5 (12.1)	76.2 (12.4)	<0.001
Fasting glucose (mmol/L)	5.7 (5.2, 6.6)	5.8 (5.3, 6.8)	5.3 (4.9, 5.6)	<0.001
Total cholesterol (mmol/L)	5.1 (1.0)	5.1 (1.0)	5.1 (1.0)	0.295
Triglycerides (mmol/L)	1.7 (1.2, 2.6)	1.8 (1.2, 2.7)	1.2 (0.9, 1.8)	<0.001
LDL-C (mmol/L)	2.8 (0.8)	2.8 (0.8)	2.7 (0.8)	0.008
Current smoking	3,274 (54.58%)	3,239 (62.35%)	35 (4.35%)	<0.001
Alcohol consumption	2,939 (48.99%)	2,894 (55.71%)	45 (5.60%)	<0.001
Hypertension	2,747 (46.32%)	2,506 (48.77%)	241 (30.43%)	<0.001
Diabetes	1,450 (24.24%)	1,370 (26.44%)	80 (9.98%)	<0.001
Dyslipidemia	3,811 (63.77%)	3,430 (66.29%)	381 (47.51%)	<0.001
Fatty liver disease	3,636 (60.68%)	3,339 (64.36%)	297 (36.94%)	<0.001
Carotid plaque	1,868 (40.77%)	1,765 (44.21%)	103 (17.46%)	<0.001
Metabolic syndrome	3,250 (54.97%)	2,973 (58.06%)	277 (35.02%)	<0.001

1 Mean (SD); Median (Q1, Q3); *n* (%); Mean ± SD for variables with skewness<1, Median [IQR] for others.

2 Welch Two Sample *t*-test; Wilcoxon rank sum test; Pearson’s Chi-squared test.

The prevalence of metabolic risk factors was substantial in this occupational cohort. Overall, 82.01% of participants were overweight or obese (BMI ≥ 24 kg/m^2^). Hypertension was present in 46.32% of the total population (48.77% in males vs. 30.43% in females, *p* < 0.001), and diabetes/elevated fasting glucose was identified in 24.24% (26.44% vs. 9.98%, *p* < 0.001). Dyslipidemia was highly prevalent at 63.77% overall (66.29% in males vs. 47.51% in females, *p* < 0.001). Fatty liver disease affected 60.68% of the cohort (64.36% of males vs. 36.94% of females, *p* < 0.001). Metabolic syndrome (MetS), defined by the 2009 harmonized criteria (≥3 of 5 components), was present in 54.97% of participants (58.06% of males vs. 35.02% of females, *p* < 0.001).

Among the 4,582 participants with valid carotid ultrasound data, the overall prevalence of carotid plaque was 40.77%. The prevalence was significantly higher in males than in females (44.21% vs. 17.46%, *p* < 0.001). Participants excluded due to missing carotid data (*n* = 1,417; 23.62%) were significantly younger than those included (mean age 42.2 vs. 50.8 years, *p* < 0.001); detailed comparisons are provided in Supplementary Table S1.

### Occupational characteristics

Table 2 and Figure 1 present the occupational characteristics from the parallel survey (*n* = 5,922). The taxi driver population was characterized by substantial occupational exposures. The majority (62.25%) had worked as taxi drivers for 20 years or more, with a mean tenure of 14.9 ± 8.0 years. Income was generally low, with 66.61% earning less than 5,000 CNY per month (<$700 USD) and only 5.14% earning more than 8,000 CNY monthly.

**Table 2:** Occupational and lifestyle characteristics of Beijing taxi drivers (*N* = 5,922).

Domain	Characteristic	*n* (%) or Mean ± SD
Demographics	Sex (male)	4,742 (86.31)
	Age, years	48.7 ± 7.7
	Married	5,255 (91.36)
	Education ≥ high school	1,136 (19.18)
	Work tenure, years	14.9 ± 8.0
Work characteristics	Years as taxi driver ≥20	3,514 (62.25)
	Monthly income <5,000 CNY	3,702 (66.61)
	Night/rotating/double shift	1,522 (25.70)
	Monthly rest days	4.0 (2.0, 5.0)
	Monthly rest days <4	1,912 (36.30)
Work-related difficulties	Meal difficulty during work	3,157 (53.31)
	Toilet access difficulty	3,445 (58.17)
	Habitual breakfast skipping	1,367 (23.08)
Sleep	Sleep duration, hours	6.8 ± 1.2
	Sleep duration <7 h	2,225 (40.78)
	Poor/very poor sleep quality	1,322 (22.32)
Lifestyle	Current smoker	2,175 (36.73)
	Current drinker	1,570 (26.51)
	Rarely exercise	1,277 (23.02)
Psychosocial factors	Job burnout score (1–7 scale)	3.4 ± 1.0
	Work stress score (1–5 scale)	3.0 ± 0.8
	Job satisfaction (satisfied/very satisfied)	1,329 (22.44)
	Turnover intention score (1–5 scale)	2.7 ± 0.8

Values are presented as *n* (%) for categorical variables and mean ± SD or median [IQR] for continuous variables.

Data source: Occupational health survey of Beijing taxi drivers, 2022–2023.

Most drivers (71.07%) worked day shifts, while 28.93% worked night, rotating, or double shifts. Work-related barriers to healthy behaviors were highly prevalent: 58.17% reported difficulty accessing restrooms during work shifts, 53.31% experienced meal-related difficulties, and 23.08% habitually skipped breakfast.

Mean sleep duration was 6.82 ± 1.16 h, and nearly one-quarter (22.32%) reported poor or very poor sleep quality. Current smoking prevalence was 36.73%. Only 16.29% reported regular physical exercise, while 21.56% rarely exercised. Moderate levels of job burnout (mean score 3.44/7) and work stress (2.95/5) were observed, and job satisfaction was low, with only 23.69% reporting being satisfied or very satisfied with their work.

Detailed tabulated and graphical occupational survey results are provided in Supplementary Tables S3–S11 and Supplementary Figures S1–S5.

### Comparison with general population

**Figure 2:**
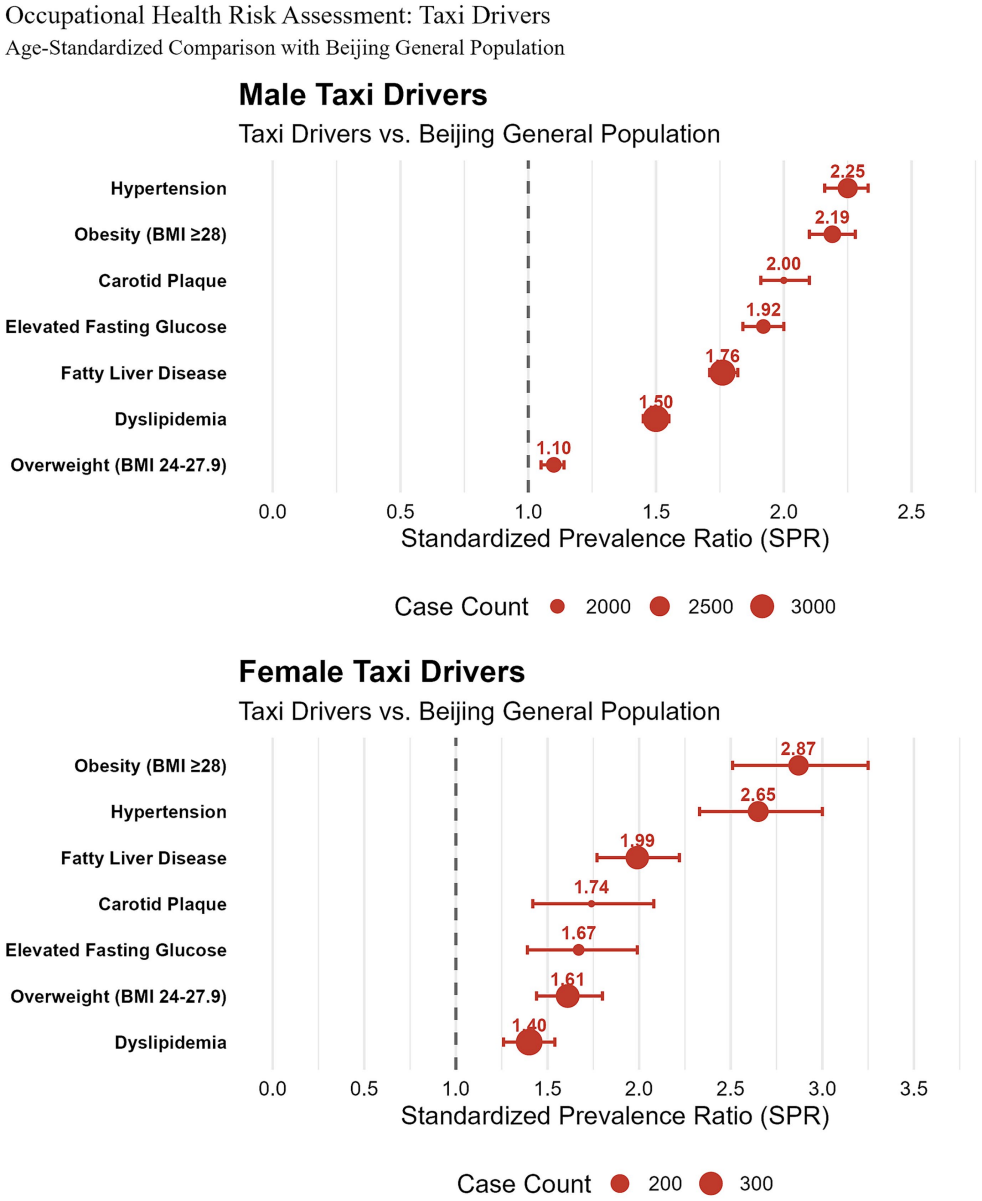
Age-standardized prevalence ratios (SPRs) for metabolic risk factors in male taxi drivers compared with Beijing general population. Forest plot showing SPRs and 95% confidence intervals for each metabolic risk factor. The vertical dashed line indicates SPR = 1.0 (no difference from general population).

Table 3 and Figure 2 present the age-standardized comparison of metabolic risk factor prevalences between male taxi drivers and the Beijing general health examination population (2022–2023). Male taxi drivers exhibited significantly elevated age-standardized prevalences across all metabolic risk factors compared with the general population (all *p* < 0.001). The most pronounced excess was observed for hypertension (SPR = 2.25, 95% CI: 2.16–2.33), followed by obesity (SPR = 2.19, 95% CI: 2.10–2.28), carotid plaque (SPR = 2.00, 95% CI: 1.91–2.10), elevated fasting glucose(SPR = 1.92, 95% CI: 1.84–2.00), fatty liver disease (SPR = 1.76, 95% CI: 1.71–1.82), dyslipidemia (SPR = 1.50, 95% CI: 1.45–1.55), and overweight (SPR = 1.10, 95% CI: 1.05–1.14). The pattern was consistent across age strata, with similar elevations observed in younger and older age groups.

**Table 3:** Comparison with general population.

Sex	Indicator	Observed cases	Expected cases	SPR (95% CI)	*p*-value
Male	Overweight (BMI 24–27.9)	2,076	1,895.0	1.10 (1.05–1.14)	<0.001
	Obesity (BMI ≥ 28)	2,241	1,022.5	2.19 (2.10–2.28)	<0.001
	Dyslipidemia	3,430	2,294.3	1.50 (1.45–1.55)	<0.001
	Elevated fasting glucose	2,007	1,046.2	1.92 (1.84–2.00)	<0.001
	Hypertension	2,506	1,116.0	2.25 (2.16–2.33)	<0.001
	Fatty liver disease	3,339	1,892.3	1.76 (1.71–1.82)	<0.001
	Carotid plaque	1,765	881.1	2.00 (1.91–2.10)	<0.001
Female	Overweight (BMI 24–27.9)	306	189.7	1.61 (1.44–1.80)	<0.001
	Obesity (BMI ≥ 28)	231	80.4	2.87 (2.51–3.25)	<0.001
	Dyslipidemia	381	272.8	1.40 (1.26–1.54)	<0.001
	Elevated fasting glucose	118	70.5	1.67 (1.39–1.99)	<0.001
	Hypertension	241	90.8	2.65 (2.33–3.00)	<0.001
	Fatty liver disease	297	149.4	1.99 (1.77–2.22)	<0.001
	Carotid plaque	103	59.3	1.74 (1.42–2.08)	<0.001

SPR, standardized prevalence ratio; SPR > 1 indicates higher prevalence in taxi driver population.

Reference data source: Beijing health examination statistical report 2022–2023.

### Independent risk factors for cardiovascular outcomes

For hypertension, independent risk factors included age (OR = 1.05 per year, 95% CI: 1.05–1.06), female sex was protective (OR = 0.66, 95% CI: 0.54–0.79), BMI (OR = 1.14 per unit, 95% CI: 1.12–1.16), and alcohol consumption (OR = 1.48, 95% CI: 1.32–1.67). Current smoking was inversely associated with hypertension after adjustment (OR = 0.81, 95% CI: 0.72–0.92).

For diabetes/elevated fasting glucose, independent associations were observed with age (OR = 1.07 per year, 95% CI: 1.06–1.08), female sex was protective (OR = 0.44, 95% CI: 0.34–0.57), BMI (OR = 1.09 per unit, 95% CI: 1.07–1.10), and current smoking (OR = 1.21, 95% CI: 1.05–1.38).

For dyslipidemia, age was not independently associated (OR = 1.00, 95% CI: 0.99–1.01, *p* = 0.641), while female sex was protective (OR = 0.64, 95% CI: 0.54–0.77), BMI was positively associated (OR = 1.11 per unit, 95% CI: 1.09–1.12), and current smoking was a risk factor (OR = 1.29, 95% CI: 1.15–1.46).

For carotid plaque, age was the strongest independent risk factor (OR = 1.29 per year, 95% CI: 1.26–1.31). The age–plaque relationship was significantly non-linear (*p* < 0.0001), with prevalence rising sharply after age 50 (from 6.1% at ages 45–50 to 50.5% at ages 50–55; Supplementary Table S12; Supplementary Figure S6). Female sex was protective (OR = 0.56, 95% CI: 0.42–0.73), and current smoking was a risk factor (OR = 1.60, 95% CI: 1.37–1.86) (Table 4).

**Table 4:** Multivariate logistic regression analysis.

Outcome	Risk factor	Adjusted OR (95% CI)	*p*-value
Hypertension	Age (per year)	**1.05 (1.05–1.06)**	**<0.001**
	Female	**0.66 (0.54–0.79)**	**<0.001**
	BMI (per unit)	**1.14 (1.12–1.16)**	**<0.001**
	Current smoking	**0.81 (0.72–0.92)**	**<0.001**
	Alcohol consumption	**1.48 (1.32–1.67)**	**<0.001**
Diabetes	Age (per year)	**1.07 (1.06–1.08)**	**<0.001**
	Female	**0.44 (0.34–0.57)**	**<0.001**
	BMI (per unit)	**1.09 (1.07–1.10)**	**<0.001**
	Current smoking	**1.21 (1.05–1.38)**	**0.007**
	Alcohol consumption	0.99 (0.87–1.13)	0.913
Dyslipidemia	Age (per year)	1.00 (0.99–1.01)	0.641
	Female	**0.64 (0.54–0.77)**	**<0.001**
	BMI (per unit)	**1.11 (1.09–1.12)**	**<0.001**
	Current smoking	**1.29 (1.15–1.46)**	**<0.001**
	Alcohol consumption	1.10 (0.98–1.24)	0.120
Carotid plaque	Age (per year)	**1.29 (1.26–1.31)**	**<0.001**
	Female	**0.56 (0.42–0.73)**	**<0.001**
	BMI (per unit)	1.00 (0.98–1.02)	0.982
	Current smoking	**1.60 (1.37–1.86)**	**<0.001**
	Alcohol consumption	1.10 (0.95–1.28)	0.213

Adjusted for Age, Gender, BMI, Smoking, and Drinking. *p*-values <0.001. Adjusted for Age, Gender, BMI, Smoking, and Drinking.

*p*-values <0.001 shown as “<0.001”. Bold values indicate statistically significant associations (*p* < 0.05).

### Association between metabolic risk clustering and carotid plaque

MetS, defined by the 2009 harmonized criteria (≥3 of 5 components), was present in 55.0% of the study population. In the primary analysis, MetS was significantly associated with carotid plaque after adjustment for age, sex, smoking, and alcohol consumption (OR = 1.28, 95% CI: 1.11–1.48, *p* < 0.001). A dose–response relationship was observed: compared with participants having 0 MetS components, those with increasing numbers of components showed incrementally higher odds of carotid plaque (P for trend <0.001; Table 5).

**Table 5:** Association between MetS components and carotid plaque.

Characteristic	OR	95% CI	*p*-value
Number of MetS components			
0 components	—	—	
1 component	1.41	0.97, 2.06	0.077
2 components	1.89	1.33, 2.71	<0.001
3 components	1.88	1.34, 2.68	<0.001
4 components	2.27	1.59, 3.27	<0.001
5 components	2.27	1.47, 3.52	<0.001
P for Trend			<0.001

CI, confidence interval; OR, odds ratio.

MetS components were defined using the 2009 Joint Interim Statement harmonized criteria. Models adjusted for age, sex, smoking, and alcohol consumption.

**Figure 3:**
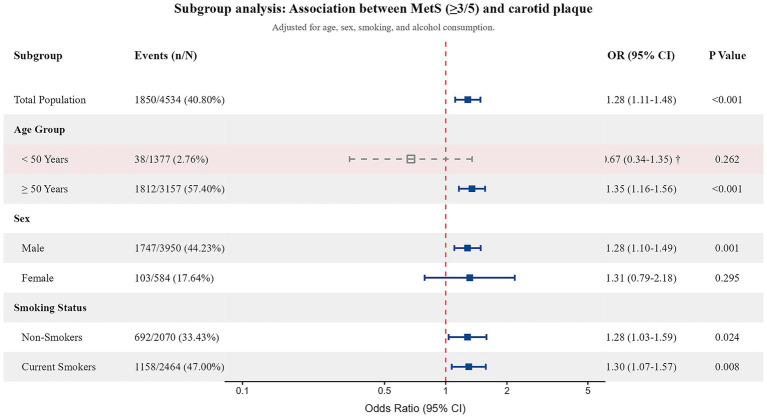
Subgroup analysis of the association between MetS and carotid plaque. The forest plot illustrates adjusted odds ratios (ORs) and 95% confidence intervals (CIs) for carotid plaque, comparing participants with MetS (≥3 of 5 components) to those without MetS. Analyses were stratified by age (<50 vs. ≥50 years), sex, and smoking status. Models were adjusted for age, sex, BMI, smoking status, and alcohol consumption, excluding the stratification variable where applicable. The vertical dashed line represents the null value (OR = 1.0). Note: The estimate for the <50 years subgroup is based on only 38 events in 1,377 participants and should be interpreted with caution due to sparse data; this subgroup is visually shaded in the figure to reflect this limitation. *p* values for interaction: Age × MetS = 0.135; Sex × MetS = 0.939; Smoking × MetS = 0.903.

Subgroup analyses demonstrated generally consistent associations of metabolic risk clustering with carotid plaque across strata (Figure 3). In age-stratified analysis, the association was significant among participants aged ≥50 years (OR = 1.35, 95% CI: 1.16–1.56, *p* < 0.001) but not among those aged <50 years (OR = 0.67, 95% CI: 0.34–1.35, *p* = 0.262), although the interaction was not statistically significant (P for interaction = 0.135). In sex-stratified analysis, the association was significant among males (OR = 1.28, 95% CI: 1.10–1.49, *p* = 0.001), whereas among females, the point estimate was similar in magnitude but did not reach statistical significance (OR = 1.31, 95% CI: 0.79–2.18, *p* = 0.295), likely due to smaller sample size (P for interaction = 0.939). When stratified by smoking status, significant associations were observed in both current smokers (OR = 1.30, 95% CI: 1.07–1.57, *p* = 0.008) and non-smokers (OR = 1.28, 95% CI: 1.03–1.59, *p* = 0.024), with no significant interaction (P for interaction = 0.903). Formal interaction-test results are presented in Supplementary Table S2.

### Sensitivity analyses

Several sensitivity analyses confirmed the robustness of the primary findings (Table 6). To address potential selection bias from missing carotid ultrasound data, inverse probability weighting (IPW) analysis was performed; the weight distribution was stable (mean = 1.07, median = 0.86, range = 0.77–21.01), and the IPW-adjusted odds ratio was 1.27 (95% CI: 1.10–1.47, *p* = 0.001), closely matching the primary estimate and indicating that differential participation in the optional carotid ultrasound is unlikely to materially bias the reported association. To further assess the influence of observations with high weights, sensitivity analyses with weight truncation at the 99th percentile and at a fixed value of 10 yielded essentially unchanged estimates (Supplementary Analysis S5). Given the high prevalence of carotid plaque, prevalence ratios were estimated using modified Poisson regression with robust standard errors; the PR for MetS was 1.12 (95% CI, 1.05–1.19, *p* < 0.001), confirming a positive association on the prevalence-ratio scale while, as expected for a common outcome, attenuating toward the null relative to the OR.

**Table 6:** Sensitivity analyses for the association between MetS and carotid plaque.

Analysis	Measure	Estimate (95% CI)	*p*-value
Primary analysis	OR	1.28 (1.11–1.48)	<0.001
Modified Poisson regression	PR	1.12 (1.05–1.19)	<0.001
Inverse probability weighting	OR	1.27 (1.10–1.47)	0.001

CI, confidence interval; IPW, Inverse Probability weighting; OR, odds ratio; PR, prevalence ratio. All models adjusted for age, sex, smoking status, and alcohol consumption. The Modified Poisson regression model used robust (sandwich) standard errors to account for the high outcome prevalence. IPW weights were derived from a logistic model predicting participation in the optional carotid ultrasound, using age, sex, BMI, smoking, drinking, hypertension, and diabetes as predictors; weight range 0.77–21.01; robust standard errors applied. A sensitivity analysis using waist-circumference-based central obesity (in place of BMI-based) yielded essentially identical results to the primary analysis (OR = 1.28, 95% CI: 1.11–1.48, *p* < 0.001) and is described in the Results.

The E-value for the point estimate (OR = 1.28) was 1.52, and for the lower confidence bound (OR = 1.11) was 1.30, indicating that an unmeasured confounder would need to be associated with both MetS and carotid plaque by a risk ratio of at least 1.52 each, above and beyond measured covariates, to fully explain the observed association. Using waist circumference instead of BMI to define central obesity yielded essentially identical results (OR = 1.28, 95% CI: 1.11–1.48, p < 0.001), indicating that the primary finding is robust to the specific definition of central obesity. Variance inflation factors for all covariates were below 1.3, indicating no multicollinearity concerns.

## Discussion

This large-scale cross-sectional study involving 5,999 taxi drivers provides comprehensive evidence of substantially elevated metabolic risk in this occupational population. Age-standardized prevalences of metabolic risk factors were substantially higher than the Beijing general population, and MetS was significantly associated with subclinical atherosclerosis. Furthermore, occupational survey data revealed high prevalences of adverse work conditions and unhealthy behaviors that provide important context for understanding these findings.

Before interpreting these findings, two features of the study warrant emphasis. First, the occupational health survey and the clinical examination data were collected from the same source population during overlapping time periods but could not be linked at the individual level, owing to the anonymized study design required for participant privacy protection. Consequently, occupational exposures are described at the population level only and were not tested as individual-level predictors of metabolic or vascular outcomes; their role in this study is contextual rather than explanatory. Second, the cross-sectional design precludes causal inference. Associations reported below should therefore be interpreted as population-level observations consistent with, but not demonstrative of, occupational causation.

The age-standardized comparison with the Beijing general population represents a key strength of this study. Among male drivers, the more than two-fold elevation in hypertension prevalence (SPR = 2.25) is particularly striking, a finding that is consistent with the high prevalence of prolonged sedentary work, irregular eating patterns, and limited opportunities for physical activity observed in the occupational survey. Similarly, the 119% excess in obesity prevalence (SPR = 2.19) and 76% excess in fatty liver disease (SPR = 1.76) underscore the substantial metabolic burden in this population. A recent meta-analysis estimated that prolonged sedentary behavior was associated with a 30% increased risk of cardiovascular events (27), a finding highly relevant to taxi drivers who spend the majority of their working hours seated.

The 100% higher prevalence of carotid plaque in males (SPR = 2.00) suggests that these metabolic derangements are accompanied by measurable subclinical atherosclerosis. This finding is consistent with previous studies documenting elevated cardiovascular mortality in professional drivers (3–5) and provides imaging-based evidence of a higher burden of subclinical atherosclerosis in this occupational population.

The parallel occupational survey provides essential context for understanding the elevated metabolic risk in taxi drivers. The prolonged sedentary nature of taxi driving, combined with irregular meal patterns (affecting more than half of drivers), work-related barriers to physical activity, and chronic sleep insufficiency, coexists with a constellation of metabolic abnormalities (28) More than one-third of drivers reported fewer than 4 rest days per month, a pattern consistent with limited recovery time and opportunities for healthy behaviors. The high prevalence of meal difficulty and toilet difficulty is consistent with practical challenges of maintaining healthy routines during extended driving shifts. Nearly one-quarter habitually skipped breakfast, a behavior that has been associated with increased cardiovascular risk in prospective studies (29).

Our findings extend and complement previous studies of taxi drivers conducted in different geographical and cultural settings. In Yazd, Iran, Rezaei Hachesu et al. (7) reported that 89.1% of taxi drivers had at least one cardiovascular risk factor, which is consistent with our finding that the vast majority of Beijing taxi drivers exhibited multiple metabolic abnormalities. However, the MetS prevalence in our study (55.0% by harmonized criteria) was notably higher than the 37.5% reported among Yazd taxi drivers using ATP III criteria (8). This difference may reflect several factors, including larger sample size, different diagnostic criteria (harmonized IDF/AHA vs. ATP III), population-specific metabolic profiles (Chinese vs. Iranian), and differences in dietary patterns and urbanization levels between Beijing and Yazd. The Yazd study also reported that 40% of drivers with MetS had an intermediate Framingham 10-year cardiovascular risk, reinforcing our observation that metabolic risk clustering in taxi drivers is associated with measurable cardiovascular consequences, as evidenced by the significant association with carotid plaque in our study (8). In Tabriz, Iran, Abedi et al. (9) found that 12.4% of taxi drivers had cardiovascular disease and highlighted the significant interplay between sleep disorders, depression, and cardiovascular risk among professional drivers. Although our study did not directly measure depression or sleep quality using validated instruments, our occupational survey revealed that 24% of Beijing taxi drivers reported poor or very poor sleep quality and mean sleep duration was only 6.82 h, paralleling the findings from Tabriz. Collectively, these cross-national comparisons suggest that the elevated metabolic and cardiovascular risk observed in taxi drivers is a consistent, global phenomenon driven by shared occupational exposures, though the magnitude varies across populations.

The high current smoking prevalence and low exercise rates further compound these occupational hazards. While individual-level linkage between clinical and survey data was not feasible due to the anonymized design, the consistent pattern of excess metabolic risk across virtually all indicators is compatible with a contribution of occupational factors, although the ecological nature of the occupational data and the cross-sectional design preclude causal conclusions.

The strong independent association between age and carotid plaque (OR = 1.29 per year) merits comment. Analysis of age-stratified prevalence revealed a markedly non-linear, threshold-like pattern: plaque prevalence was virtually zero below age 40, rose gradually to 6.1% at ages 45–50, and then increased sharply to 50.5% at ages 50–55 and 73.7% at ages 60–70 (Supplementary Table S12). Formal testing confirmed significant non-linearity (*p* < 0.0001; Supplementary Figure S6). Sensitivity analyses excluding participants aged <40 and <45 yielded per-year ORs of 1.28 and 1.24, respectively, indicating that while the zero-event young strata contributed modestly, the steep age gradient is a robust feature of the data. This pattern likely reflects two complementary factors. The first is the natural history of atherosclerosis, in which subclinical plaque accumulates gradually over decades before reaching conventional ultrasound detection thresholds. The second is ascertainment effects inherent to real-world clinical ultrasound assessment, as discussed in the Limitations. Critically, because the MetS–plaque association was adjusted for age and the dose–response analysis was conducted within the same examination protocol, these ascertainment effects do not compromise the validity of the primary association estimates reported here.

Our finding that MetS is associated with carotid plaque extends the existing literature on metabolic syndrome and subclinical atherosclerosis to this specific occupational population. A meta-analysis of population-based studies reported a pooled OR of 1.39 (95% CI: 1.23–1.57) for the association between MetS and carotid plaque (30). Our observed OR of 1.28 (95% CI: 1.11–1.48) for MetS using the harmonized IDF/ATP III definition is consistent with these estimates. The dose–response relationship, with incrementally higher odds of plaque with increasing numbers of MetS components (OR from 1.41 for 1 component to 2.27 for 5 components), supports the clinical importance of addressing multiple risk factors simultaneously.

Although the adjusted OR of 1.28 represents a modest individual-level association, its population-level implications are substantial given the high MetS prevalence (56.0%) in this cohort. Using the Levin formula, the estimated population-attributable fraction for the MetS–plaque association is approximately 13.7%, indicating that, if the association were causal, approximately one in seven cases of carotid plaque in this occupational population could be attributed to MetS. The modest per-individual effect size therefore translates into meaningful public health relevance at the population scale, underscoring the importance of population-wide preventive strategies in high-prevalence occupational groups such as taxi drivers.

Subgroup analyses showed generally consistent associations across age, sex, and smoking strata, with point estimates clustered in a narrow range (OR 1.28–1.35) and no significant interactions detected (all P for interaction > 0.1; Figure 3; detailed estimates presented in the Results). The attenuated association among participants aged <50 years likely reflects the low prevalence of carotid plaque before age 50 (only 38 events in 1,377 participants), limiting statistical power rather than indicating a true absence of effect. This observation underscores the importance of early intervention in younger workers, before atherosclerosis progresses to detectable levels.

A noteworthy observation was the inverse association between current smoking and hypertension in multivariable-adjusted models (OR = 0.81, 95% CI: 0.72–0.92). This finding is consistent with the well-documented smoking–blood pressure paradox, whereby cross-sectional studies frequently observe lower blood pressure levels in current smokers compared with non-smokers (31). This paradox is thought to be partly mediated by the lower body weight associated with smoking, and Mendelian randomization evidence suggests that smoking does not have a direct causal effect on blood pressure levels (32). Importantly, smoking was significantly associated with carotid plaque in our study (OR = 1.60, 95% CI: 1.37–1.86), consistent with its direct pro-atherogenic effects on the vascular endothelium (33). Two cross-sectional biases may further contribute to this inverse association. Taxi driving is a physically demanding occupation, and drivers who develop severe smoking-related morbidities are likely to exit the workforce, leaving the currently active smokers as a relatively healthier subset (a healthy worker effect). Similarly, tobacco-related premature morbidity and mortality may have differentially removed less resilient smokers from the sampling frame (survivorship bias). Both biases operate toward underestimating the smoking–hypertension association, and neither can be adequately addressed in a cross-sectional design. Longitudinal cohort studies that follow workers who have left the occupation would be needed to fully characterize the smoking–blood pressure relationship in this population.

This study has several strengths including the large sample size, standardized clinical assessments, age-standardized comparison with the general population, comprehensive occupational exposure characterization, and multiple sensitivity analyses confirming robustness of findings.

Several limitations warrant consideration. First, the cross-sectional design precludes causal inference regarding the observed associations between metabolic risk clustering and carotid plaque. Second, due to the anonymized design of the occupational survey, individual-level linkage with clinical data was not possible; both datasets were collected from the same population during the same period, allowing population-level but not individual-level integration of clinical and occupational data. Importantly, the occupational survey data were analyzed at the population level and were not linked to individual clinical outcomes; therefore, the occupational contextualization in this study is ecological in nature, and occupational variables were not tested as individual-level predictors of carotid plaque. Furthermore, reverse causation cannot be excluded: individuals with pre-existing metabolic conditions may preferentially enter or remain in sedentary occupations such as taxi driving, and the observed metabolic burden may partly reflect self-selection rather than occupational causation. Third, 23.6% of participants declined the optional carotid ultrasound. Those who declined were substantially younger (mean age 42.2 vs. 50.8 years, *p* < 0.001), and because carotid plaque prevalence increases sharply after age 50, the overall crude plaque prevalence of 40.77% likely overestimates the prevalence that would have been observed in the full cohort. To address this, we applied inverse probability weighting, which yielded a MetS–plaque OR of 1.27 (95% CI: 1.10–1.47), essentially identical to the primary estimate (OR = 1.28). This consistency provides strong evidence that differential participation is unlikely to bias the association between MetS and carotid plaque. However, IPW mitigates but does not fully eliminate bias in the presence of substantial baseline differences, and residual bias cannot be excluded, particularly bias affecting absolute prevalence rather than association estimates. Readers should therefore interpret the 40.77% crude plaque prevalence figure as an upper-bound estimate for this cohort. Fourth, we lacked data on dietary intake beyond meal patterns, and physical activity was self-reported. Fifth, we did not assess mental health indicators such as depression or anxiety using validated instruments, which have been shown to be prevalent among taxi drivers and may contribute to cardiovascular risk. Sixth, the reference population data from the Beijing Health Examination Annual Report may include individuals with varying health-seeking behaviors, potentially affecting the standardized prevalence comparisons, though the use of age stratification mitigates this concern to some extent. These comparisons should therefore not be interpreted as fully representative of the entire Beijing general population. Seventh, participants were recruited from a voluntary labor-union-sponsored health program, which may introduce participation bias toward more health-conscious drivers; even within the Beijing taxi driver workforce, our cohort may not fully represent drivers who are not union members or who declined the health examination. Further, the generalizability of our findings may be limited to urban Chinese taxi drivers, and caution is needed when extrapolating to taxi driver populations in other countries or to rural settings. Eighth, this study used carotid ultrasound data from routine health examinations rather than from a prospective research protocol. As is customary in high-volume examination centers, carotid ultrasonography was performed by the examination center’s pool of certified sonographers on rotation, following standardized plaque detection criteria but without formal blinding to participant age or prospective assessment of interobserver agreement. Consistent with this real-world design, the age-stratified crude prevalence of carotid plaque increased sharply between the 45–49 and 50–54 age strata (from 6.1% to 50.5%), a gradient that exceeds what would be expected from the natural progression of atherosclerosis alone and likely reflects operator-level ascertainment effects, in which subtle or borderline plaques may be more readily reported in older participants whose higher pre-test probability prompts closer inspection. Importantly, such ascertainment effects would be expected to influence absolute prevalence estimates, with younger strata in particular potentially being underestimated. However, they should not bias within-cohort association estimates that are adjusted for age, including the primary MetS–plaque association and the age-standardized prevalence ratios. The use of objective Mannheim consensus criteria and the large pool of trained sonographers are expected to further reduce, though not eliminate, inter-operator variability. Future studies employing standardized research-grade protocols with blinded assessment and formal inter-rater reliability testing would help refine prevalence estimates in younger workers. Ninth, the study involved multiple regression models, subgroup analyses, and interaction tests without formal adjustment for multiple comparisons. Secondary and subgroup findings should therefore be interpreted with appropriate caution as hypothesis-generating rather than confirmatory.

The E-value analysis provides a quantitative assessment of potential unmeasured confounding. The E-value of 1.52 for the primary point estimate (OR = 1.28) indicates that an unmeasured confounder would need to be associated with both MetS and carotid plaque by a risk ratio of at least 1.52 each, above and beyond measured covariates, to fully explain the observed association. While this threshold is moderate rather than high, the major established risk factors for carotid atherosclerosis, namely age, sex, smoking, and alcohol consumption, were already adjusted for in our models. Residual confounding from unmeasured factors (e.g., family history, genetic predisposition) would need to meet this threshold to fully explain the findings, which we consider unlikely in the context of the well-documented biological link between metabolic dysfunction and atherogenesis.

Our findings have important implications for occupational health policy in this population. The barriers identified in our occupational survey—insufficient rest (over one-third reported fewer than 4 monthly rest days), meal access difficulties (53%), toilet access difficulties (58%), and high smoking prevalence (37%)—point to specific intervention targets that require both workplace-level and regulatory-level action. At the workplace level, evidence from comparable populations supports the feasibility of taxi-driver-targeted health programs. Gany et al. (34) have demonstrated through the Step On It! worksite initiative that taxi-stand-based programs can substantially improve healthcare access, primary care linkage, and cardiovascular risk awareness in this occupational group; their subsequent HAILL randomized controlled trial provided further evidence that cluster-based interventions delivered through occupational structures can improve primary care uptake in for-hire vehicle drivers (35). Pedometer-based physical activity interventions and culturally tailored smoking cessation programs delivered at taxi stands have shown promising signals in similar populations (36). At the regulatory level, China’s existing labor framework permits flexible work-hour regimes for taxi drivers under State Council Circular Laobufa No. 503 (1994), which—while necessary for the operational nature of taxi work—creates a context in which the standard 8-h-day / 40-h-week protections are not directly enforced. Strengthening minimum monthly rest-day standards, requiring designated rest and meal facilities at major taxi dispatch points, and integrating annual cardiovascular screening into union-sponsored health programs (as in the present cohort) represent feasible regulatory and structural levers. Given the substantially elevated cardiovascular risk documented here, particularly among older male drivers, enhanced cardiovascular screening with structured follow-up for those with multiple metabolic risk factors should be considered a priority within this population.

The substantially elevated metabolic risk in this population also supports consideration of enhanced cardiovascular screening, particularly for older drivers with multiple risk factors. Early detection of subclinical atherosclerosis may enable more intensive preventive interventions before clinical events occur.

## Conclusion

Chinese taxi drivers bear a substantially elevated burden of metabolic disorders compared with the general population, with age-standardized prevalences of obesity, hypertension, fatty liver disease, and carotid plaque 1.1- to 2.25-fold higher than expected in males. MetS is significantly associated with subclinical atherosclerosis (OR = 1.28, 95% CI: 1.11–1.48), with a clear dose–response relationship. Subgroup analyses confirmed the consistency of this association across age, sex, and smoking strata, with no significant interactions detected. The high prevalence of adverse occupational exposures—including insufficient rest, meal difficulties, sedentary work, and smoking—provides important context for these findings. Targeted occupational health interventions addressing both lifestyle factors and structural work conditions in this high-risk population may be warranted.

## Data Availability

The raw data supporting the conclusions of this article will be made available by the authors, without undue reservation.
