# Supplementary material for: Metabolic syndrome and carotid plaque in Chinese taxi drivers: a cross-sectional study of 5,999 participants
Source: Front Public Health. 2026 Jun 8;14:1812128. doi: 10.3389/fpubh.2026.1812128 (PMC13284099; doi:10.3389/fpubh.2026.1812128)

**Supplementary Materials**

**Supplementary Methods**

**S1. Handling of Missing Carotid Ultrasound Outcomes**

Carotid ultrasonography was optional in the annual health examination, and 1,417 of 5,999 participants (23.6%) had missing plaque outcomes due to non-participation. To assess potential selection bias, inverse probability weighting (IPW) was applied. The probability of having complete carotid ultrasound data was estimated using logistic regression based on baseline covariates. The primary multivariable model for carotid plaque was then refitted using IPW. Weight-distribution diagnostics are shown in Supplementary Figure S1.

**S2. Restricted Cubic Spline Analysis**

To evaluate the dose-response relationship between metabolic risk burden and carotid plaque, restricted cubic spline models were fitted with the number of metabolic risk factors as a continuous exposure. Models were adjusted for age, sex, smoking status, and alcohol consumption. The spline curve is presented in Supplementary Figure S2.

**S3. E-value Analysis**

To quantify robustness to unmeasured confounding, E-values were calculated for the primary effect estimate and its confidence bound for the association between metabolic risk clustering and carotid plaque.

**S4. Interaction and Subgroup Analyses**

Effect modification was evaluated by including interaction terms between metabolic risk clustering and age group, sex, and smoking status. Formal interaction-test results are provided in Supplementary Table S2.

**Supplementary** **Analysis S5. IPW Weight Truncation Sensitivity Analysis**

Stabilized inverse probability weights for the carotid ultrasound participation model had a maximum value of 21.01. To assess whether this small number of observations with extreme propensity scores unduly influenced the primary estimate, we repeated the IPW analysis after truncating weights at two pre-specified thresholds: (i) the 99th percentile of the weight distribution (4.28), and (ii) a fixed value of 10. The MetS–plaque association was essentially unchanged under both truncation schemes (truncated at 99th percentile: OR=1.27, 95% CI: 1.10–1.47; truncated at 10: OR=1.27, 95% CI: 1.10–1.47), matching the untruncated IPW estimate to three decimal places. This indicates that the primary IPW-adjusted association estimate is not driven by a small number of observations with extreme weights. Robust (sandwich) standard errors were applied in all weighted models.

**Supplementary Tables**

**Supplementary Table S1. Comparison of baseline characteristics between participants with and without carotid ultrasound data.**

| Characteristic | Included (n = 4,582) | Excluded (n = 1,417) | P-value |
| --- | --- | --- | --- |
| Age, years | 50.8 ± 6.8 | 42.2 ± 7.1 | <0.001 |
| Sex, Female | 588 (12.8%) | 216 (15.2%) | <0.001 |
| BMI, kg/m² | 27.5 ± 4.1 | 27.7 ± 4.4 | 0.089 |
| Waist circumference, cm | 93.2 ± 10.5 | 92.5 ± 11.2 | 0.034 |
| Systolic BP, mmHg | 132.5 ± 17.9 | 130.1 ± 18.6 | <0.001 |
| Diastolic BP, mmHg | 82.8 ± 12.3 | 81.5 ± 12.7 | 0.001 |
| Fasting glucose, mmol/L | 5.8 (5.3, 6.8) | 5.6 (5.1, 6.3) | <0.001 |
| Total cholesterol, mmol/L | 5.1 ± 1.0 | 5.0 ± 0.9 | 0.002 |
| Triglycerides, mmol/L | 1.7 (1.2, 2.6) | 1.7 (1.1, 2.5) | 0.124 |
| LDL-C, mmol/L | 2.8 ± 0.8 | 2.7 ± 0.8 | 0.001 |
| HDL-C, mmol/L | 1.3 ± 0.3 | 1.3 ± 0.3 | 0.456 |
| Current smoking | 2,509 (54.8%) | 765 (54.0%) | 0.615 |
| Alcohol consumption | 2,248 (49.1%) | 691 (48.8%) | 0.852 |
| Hypertension | 758 (16.5%) | 184 (13.0%) | 0.001 |
| Diabetes | 387 (8.4%) | 77 (5.4%) | <0.001 |
| Dyslipidemia | 102 (2.2%) | 21 (1.5%) | 0.087 |
| Overweight/Obesity | 3,834 (83.7%) | 1,152 (81.3%) | 0.038 |
| Fatty liver disease | 2,856 (62.3%) | 780 (55.0%) | <0.001 |
| Number of metabolic risk factors |  |  | <0.001 |
| 0 | 572 (12.5%) | 246 (17.4%) |  |
| 1 | 2,145 (46.8%) | 712 (50.2%) |  |
| 2 | 1,432 (31.3%) | 368 (26.0%) |  |
| ≥3 | 433 (9.4%) | 91 (6.4%) |  |

**Supplementary Table S2. Interaction tests for the association between MetS and carotid plaque.**

| Stratification Variable | P for Interaction |
| --- | --- |
| Age (<50 vs ≥50) | 0.135 |
| Sex (Male vs Female) | 0.939 |
| Smoking (Yes vs No) | 0.903 |

**Supplementary Table S3. Prevalence of work-related difficulties in the occupational survey.**

| Work-related Difficulty | Prevalence (%) |
| --- | --- |
| Meal difficulty | 53.3 |
| Toilet difficulty | 58.2 |
| No breakfast habit | 23.1 |

**Supplementary Table S4. Sleep status of Beijing taxi drivers in the occupational survey.**

| Characteristic | N = 5,922 1 |
| --- | --- |
| Self-rated sleep quality |  |
| Very good | 1,112 (20%) |
| Good | 3,074 (56%) |
| Poor | 1,049 (19%) |
| Very poor | 273 (5.0%) |
| Daily sleep duration (hours) | 6.82 ± 1.16 |
| 1 n (%); Mean ± SD |  |

**Supplementary Table S5. Lifestyle factors in the occupational survey.**

| Characteristic | N = 5,922 1 |
| --- | --- |
| Smoking status |  |
| Daily | 214 (3.8%) |
| Former | 2,575 (46%) |
| Frequent | 908 (16%) |
| Never | 853 (15%) |
| Occasional | 1,053 (19%) |
| Drinking status |  |
| Former | 2,940 (57%) |
| Frequent | 315 (6.1%) |
| Never | 664 (13%) |
| Occasional | 1,255 (24%) |
| Physical exercise |  |
| Occasionally | 3,367 (61%) |
| Rarely | 1,277 (23%) |
| Regularly | 904 (16%) |
| 1 n (%) |  |

**Supplementary Table S6. Prevalence of unhealthy lifestyle behaviors.**

| Unhealthy Behavior | Prevalence (%) |
| --- | --- |
| Current smoking | 36.7 |
| Frequent drinking | 5.3 |
| Rarely exercise | 21.6 |

**Supplementary Table S7. Job burnout assessment (MBI dimensions and total score).**

| Characteristic | N = 5,922 1 |
| --- | --- |
| Emotional exhaustion (1-7) | 3.43 ± 1.47 |
| Depersonalization (1-7) | 3.00 ± 1.55 |
| Reduced personal accomplishment (1-7) | 3.89 ± 1.58 |
| Overall burnout score | 3.44 ± 0.97 |
| 1 Mean ± SD |  |

**Supplementary Table S8. Work stress assessment (domain-specific and overall scores).**

| Characteristic | N = 5,922 1 |
| --- | --- |
| Workload stress | 3.24 ± 0.92 |
| Role ambiguity stress | 2.76 ± 1.01 |
| Career development stress | 3.01 ± 1.01 |
| Interpersonal stress | 2.78 ± 0.98 |
| Organizational stress | 2.95 ± 0.97 |
| Work-family conflict | 2.79 ± 0.98 |
| Overall stress score | 2.95 ± 0.83 |
| 1 Mean ± SD |  |

**Supplementary Table S9. Job identity, turnover intention, and job satisfaction.**

| Characteristic | N = 5,922 1 |
| --- | --- |
| Job identity score (1-5) | 3.02 ± 0.83 |
| Turnover intention score (1-5) | 2.66 ± 0.82 |
| Job satisfaction |  |
| Very dissatisfied | 419 (7.5%) |
| Dissatisfied | 1,189 (21%) |
| Neutral | 2,675 (48%) |
| Satisfied | 795 (14%) |
| Very satisfied | 534 (9.5%) |
| 1 Mean ± SD; n (%) |  |

**Supplementary Table S10. Occupational and lifestyle characteristics stratified by sex.**

| Characteristic | Male N = 4,742 1 | Female N = 752 1 | p-value 2 |
| --- | --- | --- | --- |
| Age (years) | 49.2 ± 7.6 | 45.5 ± 7.8 | <0.001 |
| Work tenure (years) | 15.4 ± 8.0 | 11.7 ± 7.3 | <0.001 |
| Monthly income |  |  | <0.001 |
| <5,000 CNY | 3,063 (68%) | 409 (58%) |  |
| 5,000-8,000 CNY | 1,203 (27%) | 252 (36%) |  |
| 8,000-10,000 CNY | 173 (3.9%) | 38 (5.4%) |  |
| >10,000 CNY | 46 (1.0%) | 8 (1.1%) |  |
| Work schedule |  |  | <0.001 |
| Day shift | 2,934 (69%) | 574 (85%) |  |
| Night shift | 356 (8.3%) | 21 (3.1%) |  |
| Rotating shift | 404 (9.5%) | 52 (7.7%) |  |
| Double shift | 573 (13%) | 30 (4.4%) |  |
| Monthly rest days | 4.0 ± 2.7 | 4.4 ± 2.5 | <0.001 |
| Sleep duration (hours) | 6.8 ± 1.2 | 6.9 ± 1.1 | 0.009 |
| Poor sleep quality | 1,104 (23%) | 145 (19%) | 0.015 |
| Meal difficulty | 2,641 (56%) | 322 (43%) | <0.001 |
| Toilet difficulty | 2,893 (61%) | 362 (48%) | <0.001 |
| Current smoking | 1,976 (42%) | 37 (4.9%) | <0.001 |
| Rarely exercise | 1,070 (23%) | 130 (17%) | 0.001 |
| Burnout score | 3.5 ± 1.0 | 3.2 ± 0.9 | <0.001 |
| Work stress score | 3.0 ± 0.8 | 2.8 ± 0.7 | <0.001 |
| Job identity score | 3.0 ± 0.8 | 3.3 ± 0.8 | <0.001 |
| 1 Mean ± SD; n (%) |  |  |  |
| 2 Wilcoxon rank sum test; Pearson’s Chi-squared test |  |  |  |

**Supplementary Table S11. Summary of key occupational survey findings.**

| Indicator | Value |
| --- | --- |
| Total sample size | 5,922 |
| Male proportion | 80.1% |
| Mean age | 48.7 years |
| Mean work tenure | 14.9 years |
| Monthly income <5,000 CNY | 62.5% |
| Monthly rest days <4 | 36.3% |
| Meal difficulty | 53.3% |
| Toilet difficulty | 58.2% |
| No breakfast habit | 23.1% |
| Poor/very poor sleep | 22.3% |
| Mean sleep duration | 6.8 hours |
| Current smoking | 36.7% |
| Rarely exercise | 21.6% |
| Burnout score (1-7) | 3.44 |
| Work stress score (1-5) | 2.95 |
| Job identity score (1-5) | 3.02 |
| Satisfied/Very satisfied | 22.4% |

**Supplementary Table S12. Age-stratified carotid plaque prevalence**

| **Age Group** | **N** | **Plaque n** | **Prevalence (%)** |
| --- | --- | --- | --- |
| [20, 30) | 43 | 0 | 0.0 |
| [30, 40) | 309 | 0 | 0.0 |
| [40, 45) | 431 | 1 | 0.2 |
| [45, 50) | 611 | 37 | 6.1 |
| [50, 55) | 1,734 | 875 | 50.5 |
| [55, 60) | 1,302 | 843 | 64.7 |
| [60, 70) | 152 | 112 | 73.7 |

**Supplementary Figures**

**Supplementary Figure S1. Prevalence of work-related difficulties.**


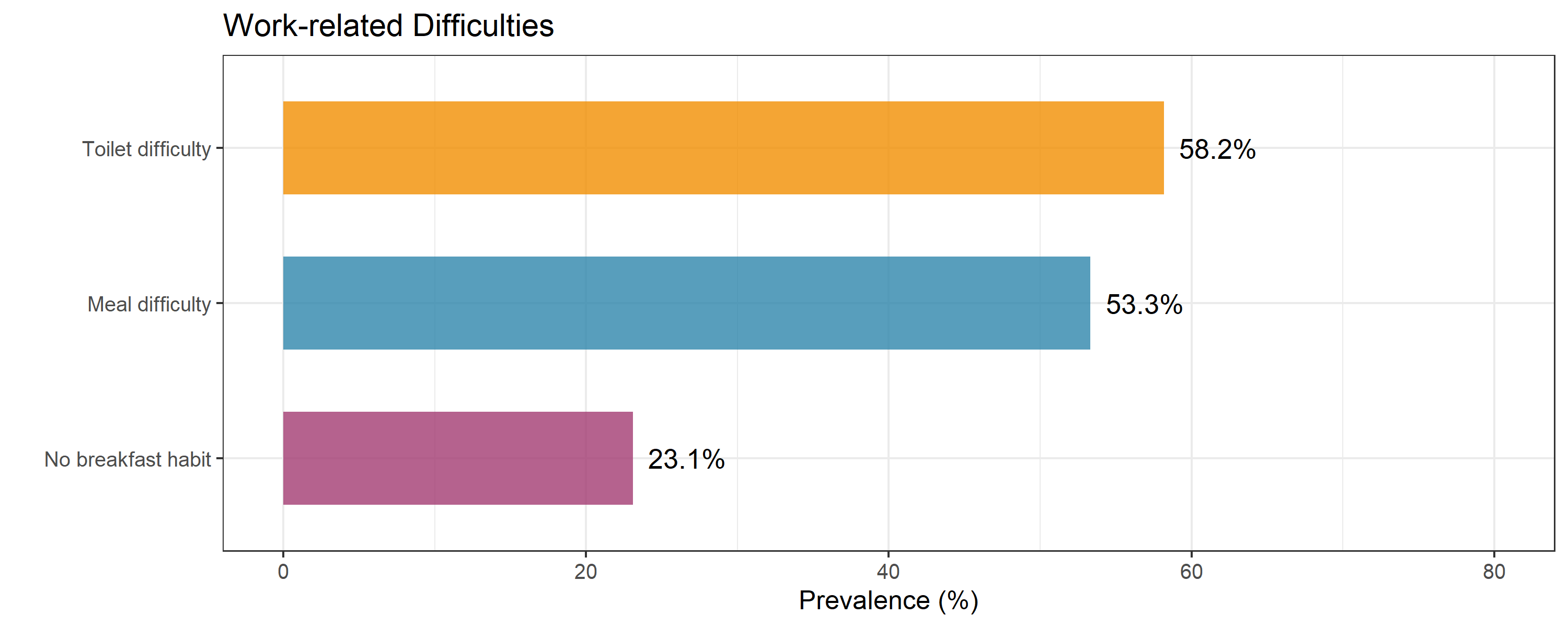


Bar chart of meal-access difficulty, restroom-access difficulty, and breakfast skipping.

**Supplementary Figure S2. Sleep quality and sleep-duration distribution.**


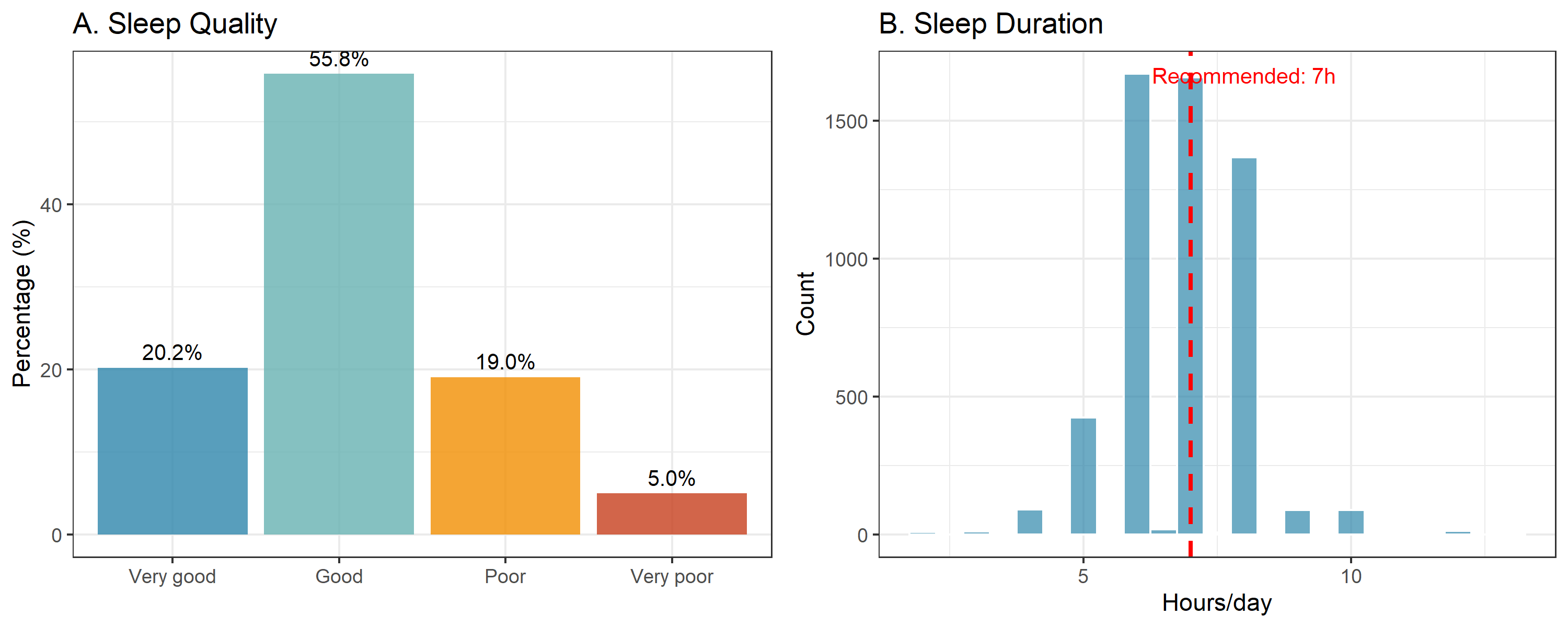


Graphical summary of self-rated sleep quality and sleep duration.

**Supplementary Figure S3. Prevalence of unhealthy lifestyle behaviors.**


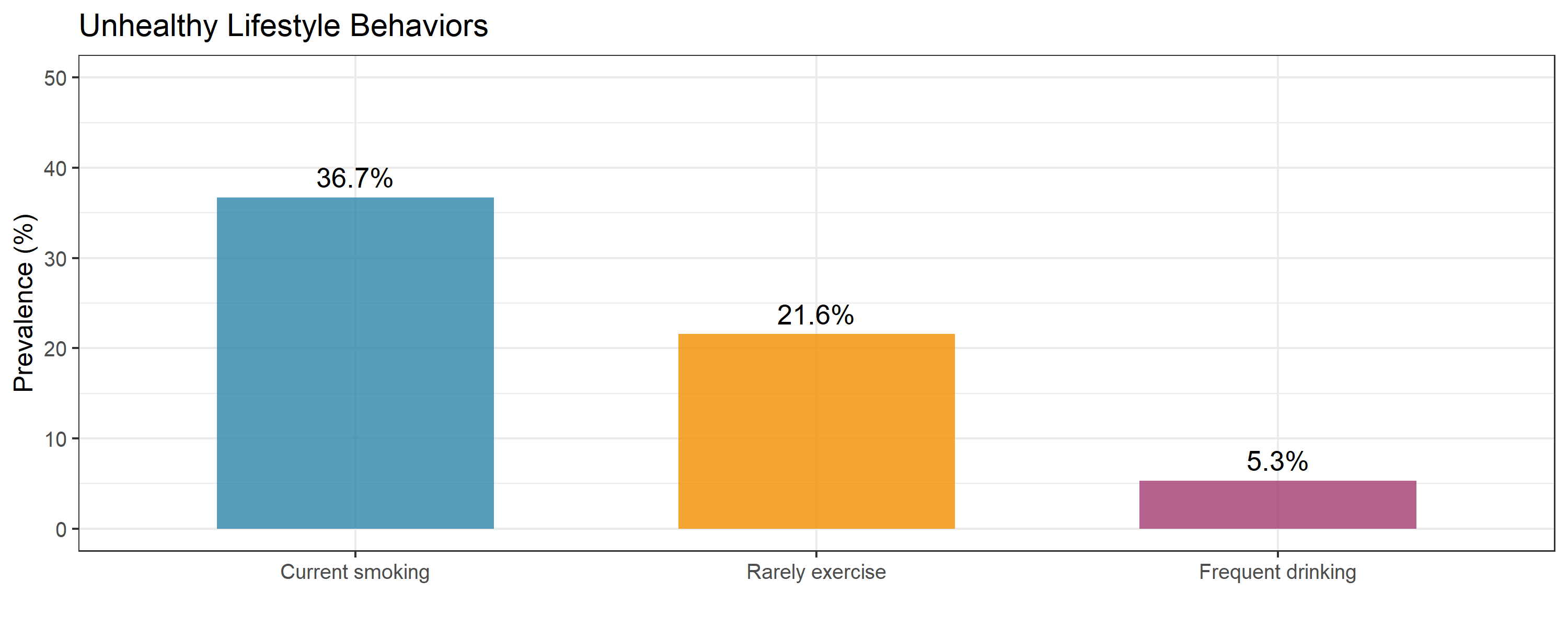


Graphical summary of current smoking, alcohol use pattern, and low physical activity.

**Supplementary Figure S4. Work stress profile by dimension.**


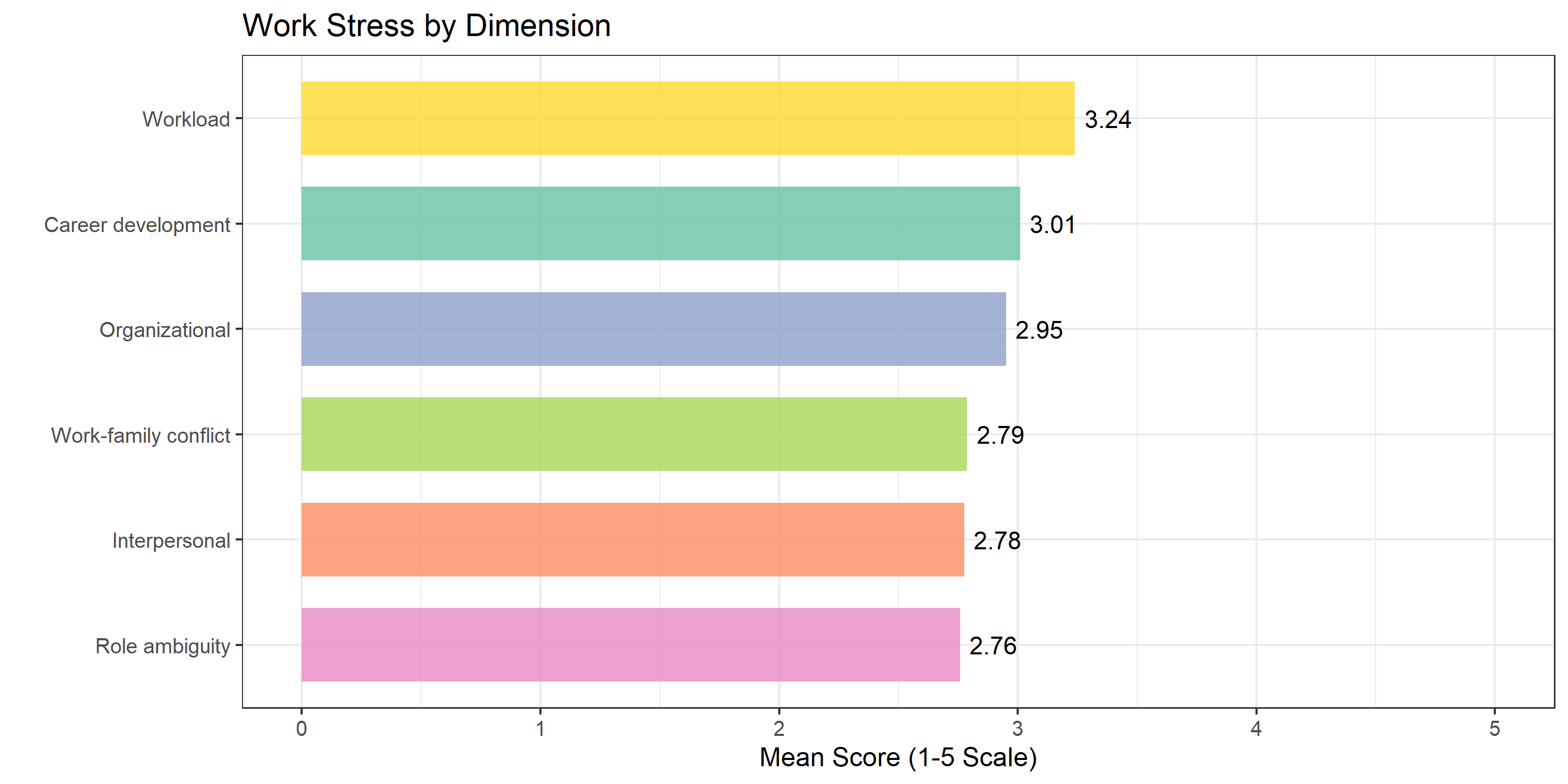


Domain-level stress distribution (workload, role ambiguity, career development, interpersonal, organizational, and work-family conflict).

**Supplementary Figure S5. Distribution of job satisfaction.**


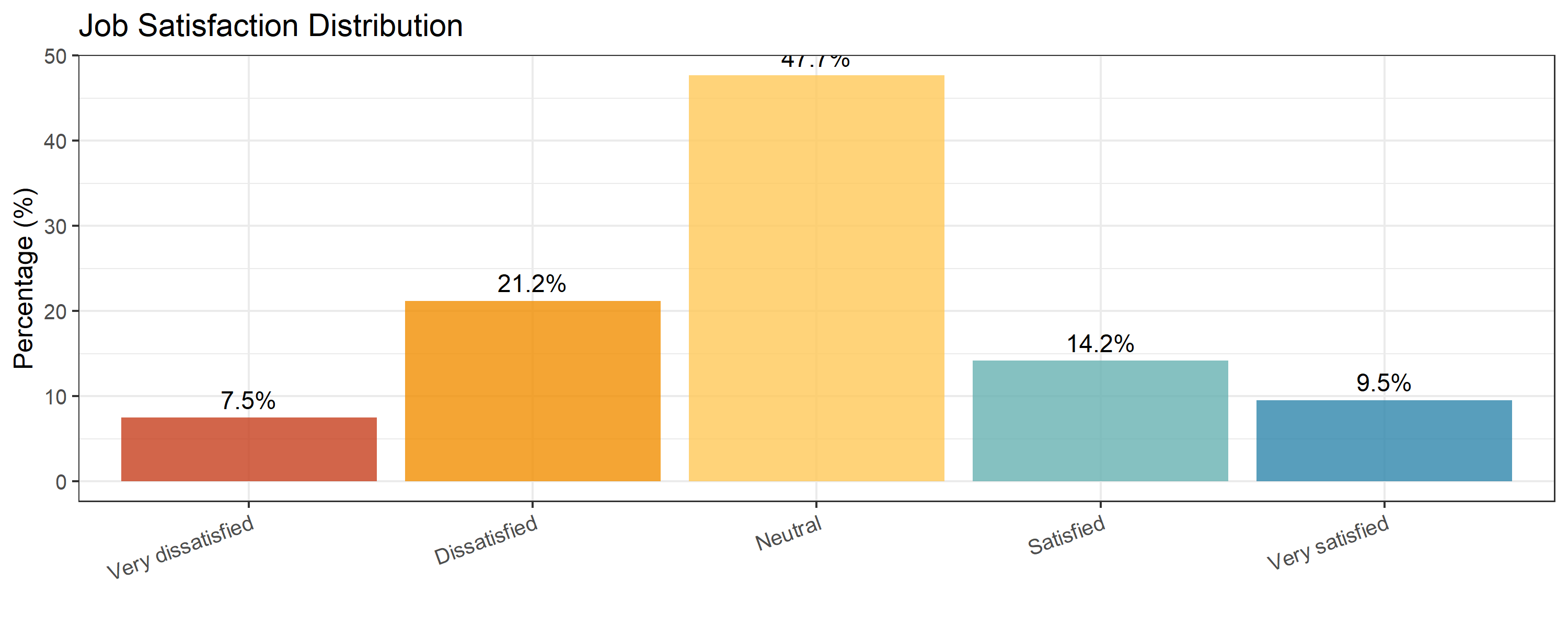


Distribution of satisfaction categories from very dissatisfied to very satisfied.

**Supplementary Figure S6. The non-linear age–plaque relationship**


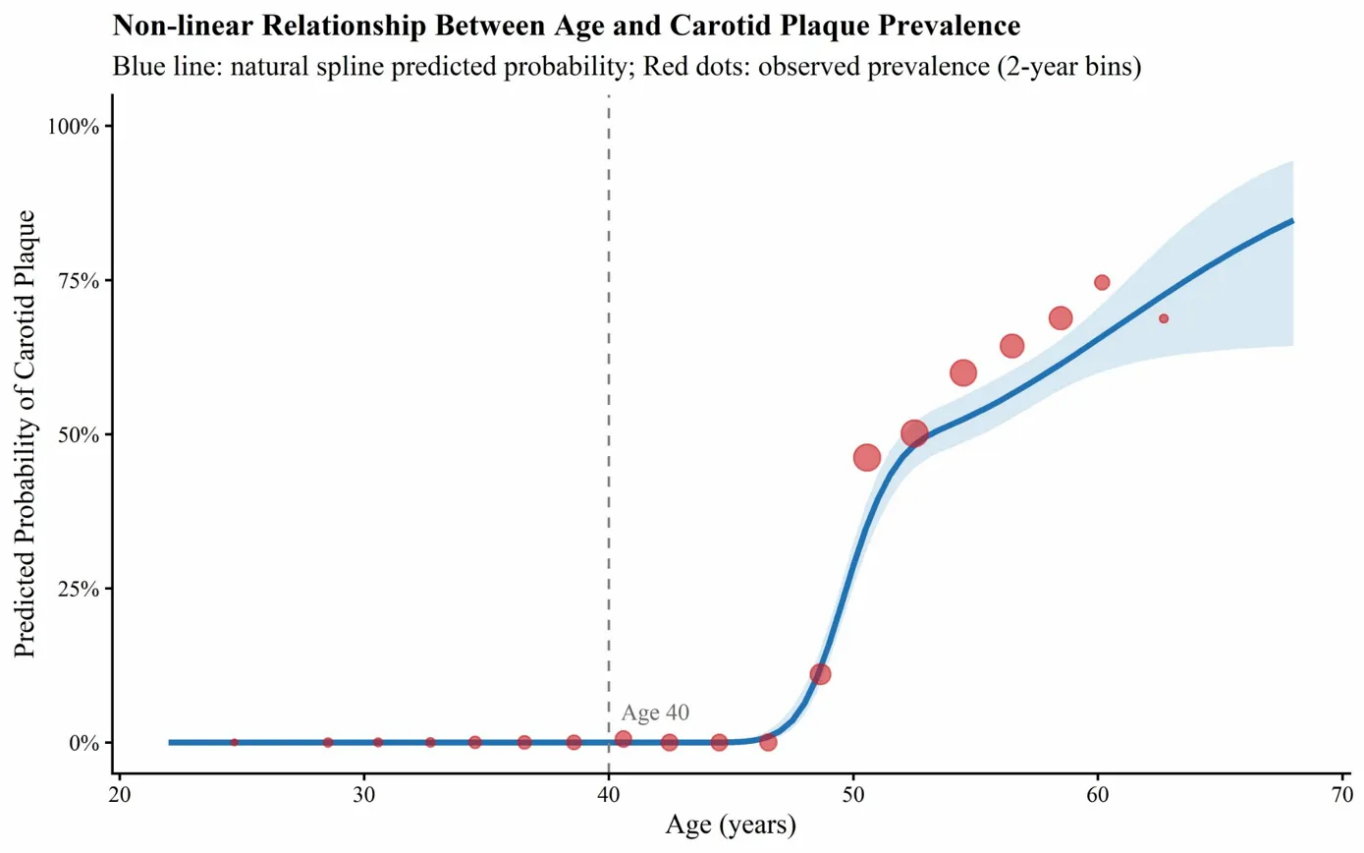

Supplement: Supplementary file 1 [file Data_Sheet_1.DOCX]
